# Tumor Progression Locus 2 (Tpl2) Deficiency Does Not Protect against Obesity-Induced Metabolic Disease

**DOI:** 10.1371/journal.pone.0039100

**Published:** 2012-06-11

**Authors:** Graeme I. Lancaster, Greg M. Kowalski, Emma Estevez, Michael J. Kraakman, George Grigoriadis, Mark A. Febbraio, Steve Gerondakis, Ashish Banerjee

**Affiliations:** 1 Cellular and Molecular Metabolism Laboratory, BakerIDI Heart and Diabetes Institute, Melbourne, Australia; 2 Department of Biochemistry and Molecular Biology, Monash University, Melbourne, Australia; 3 Intracellular Signalling and Gene Expression Laboratory, Burnet Institute, Melbourne, Australia; 4 Department of Clinical Haematology, Central Clinical School, Monash University, Melbourne, Australia; National Institute of Agronomic Research, France

## Abstract

Obesity is associated with a state of chronic low grade inflammation that plays an important role in the development of insulin resistance. Tumor progression locus 2 (Tpl2) is a serine/threonine mitogen activated protein kinase kinase kinase (MAP3K) involved in regulating responses to specific inflammatory stimuli. Here we have used mice lacking Tpl2 to examine its role in obesity-associated insulin resistance. Wild type (*wt*) and *tpl2^−/−^* mice accumulated comparable amounts of fat and lean mass when fed either a standard chow diet or two different high fat (HF) diets containing either 42% or 59% of energy content derived from fat. No differences in glucose tolerance were observed between *wt* and *tpl2^−/−^* mice on any of these diets. Insulin tolerance was similar on both standard chow and 42% HF diets, but was slightly impaired in *tpl2^−/−^* mice fed the 59% HFD. While gene expression markers of macrophage recruitment and inflammation were increased in the white adipose tissue of HF fed mice compared with standard chow fed mice, no differences were observed between *wt* and *tpl2^−/−^* mice. Finally, a HF diet did not increase Tpl2 expression nor did it activate Extracellular Signal-Regulated Kinase 1/2 (ERK1/2), the MAPK downstream of Tpl2. These findings argue that Tpl2 does not play a non-redundant role in obesity-associated metabolic dysfunction.

## Introduction

Obesity is associated with a state of chronic low grade inflammation that is believed to be important in the development of insulin resistance [1]. Research over the last decade has identified numerous events critical to the induction of inflammation in models of nutrient oversupply [1]. The accumulation of particular lipids [2], [3], the generation of reactive oxygen species [4], organelle stress [5], [6] and the activation of lipid-responsive transmembrane receptors [7], [8] are amongst a multitude of stimuli that have been suggested to link excess nutrient availability to the induction of inflammation. However, regardless of the precise mechanisms by which nutrient-mediated inflammation is initiated, a commonality exists in both the signaling pathways that are activated and the gene products that are up-regulated [1].

Two key intracellular signalling cascades that promote inflammation in response to a wide array of environmental stimuli, including nutrient oversupply, are NF-κB and the Mitogen-Activated Protein Kinases (MAPK). Consistent with the importance of these pathways in metabolic disease, conditional inactivation of the Inhibitor of kappa-B kinase β (IKKβ), the key activator of the classical NF-κB pathway, and the MAPK c-Jun N-terminal kinase (JNK) in metabolically important sites including adipose tissue [9], skeletal muscle [10] and liver [11] as well as in immune and inflammatory mediators like macrophages [11], prevents obesity-associated inflammation and protect against insulin resistance. Activation of the NF-κB and Jun transcription factors by IKKβ and JNK which leads to the up-regulation of the pro-inflammatory genes *tnf, il6*, *il1β* and *ccl2* is central to this process [1]. MAP kinases activated by obesity may also target key components of the insulin signaling cascade, resulting in insulin resistance [1].

Tumor progression locus 2 (Tpl2) also referred to as COT (Cancer Osaka Thyroid), is a serine threonine mitogen activated protein kinase kinase kinase [12] highly expressed in hemopoietic cells that specifically activates ERK 1 and 2 via the MAP kinase kinases MKK1/2 [13]. Inactive Tpl2 is part of a complex with A20-binding inhibitor of NF-κB-2 (ABIN2) and p105 NF-κB1, the precursor for the p50 NF-κB1 transcription factor. Tpl2 is activated by multiple pro-inflammatory stimuli including lipopolysaccharide (LPS), tumour necrosis factor α (TNFα) and IL-1β [14], [15] via a multi-step process initiated by the IKKβ catalysed phosphorylation dependent degradation of p105 [16], [17]. Consistent with the importance of Tpl2 in inflammatory responses, *tpl2^−/−^* mice are resistant to lipopolysaccharide (LPS)/D-galactosamine-induced endotoxic shock, an effect due to an ERK-dependent post-transcriptional regulation of TNFα [12]. The role of Tpl2 in regulating ERK-dependent inflammatory processes prompted two recent studies that investigated the role of Tpl2 in obesity-associated metabolic disorders [18], [19]. These reports show that loss of Tpl2 function prevents high fat (HF) diet-induced adipose tissue and liver inflammation plus HF diet-induced insulin resistance, the later potentially via a reduction in ERK-mediated insulin receptor substrate 1 (IRS1) serine phosphorylation. Our long standing interests in the role of Tpl2 in inflammation [13], [16], [20] and also inflammation in diet-induced metabolic disease [21]–[23], prompted an independent examination of its potential role in mediating the deleterious metabolic responses induced by a HF diet. Using a mutant mouse model lacking Tpl2, we have failed to find any significant role for Tpl2 in regulating obesity-associated metabolic disorders.

## Results

Male Tpl2-deficient (*tpl2^−/−^*) and *wt* mice were fed either a high fat (42% calories by energy content derived from fat) or standard laboratory chow (8% calories by energy content derived from fat) diet for 16 weeks, commencing at 8 weeks of age. While both standard chow and HF-fed *tpl2^−/−^* mice, plus the *wt* standard chow fed mice contain 8 mice per group, in the *wt* HFD group one mouse did not gain any fat mass during the 16 week HF diet and was excluded from all subsequent analysis. Data from this group therefore represents 7 mice.

**Figure 1:**
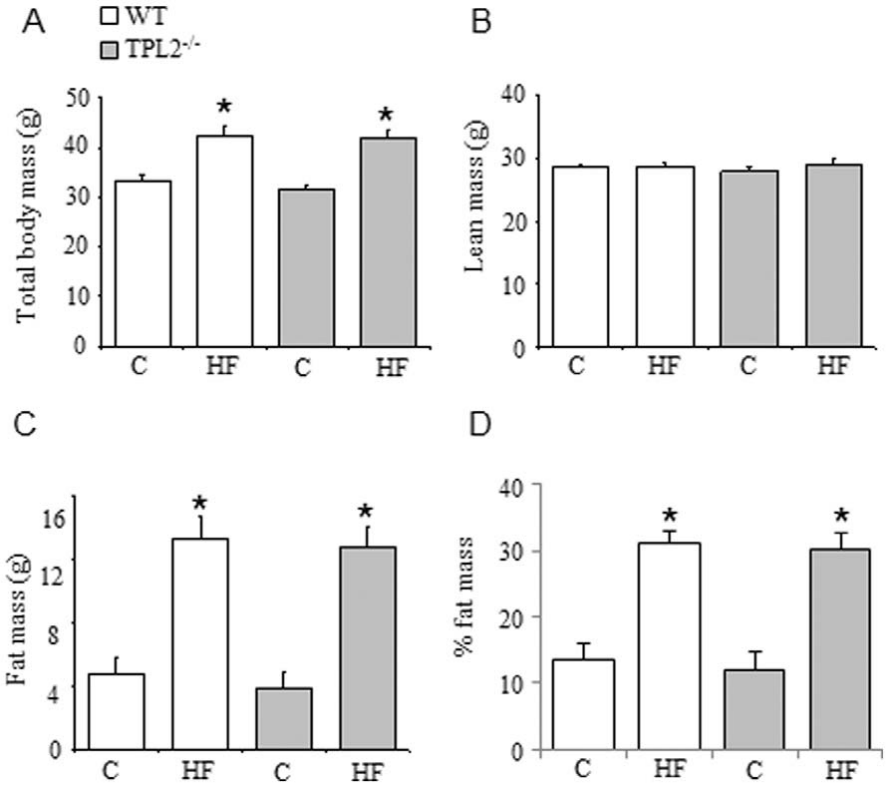
*wt* and *tpl2^−/−^* mice were fed either standard chow or high fat diets for 16 weeks. (A) Total body mass. (B and C) Lean and fat mass were determined by Echo MRI. (D) Percentage fat mass. Data presented are mean ± SEM. All groups are n = 8 with the exception of the HF fed *wt* group which is n = 7. * denotes statistically significant dietary main effect between standard chow (C) and high fat (HF) diets.

**Figure 2:**
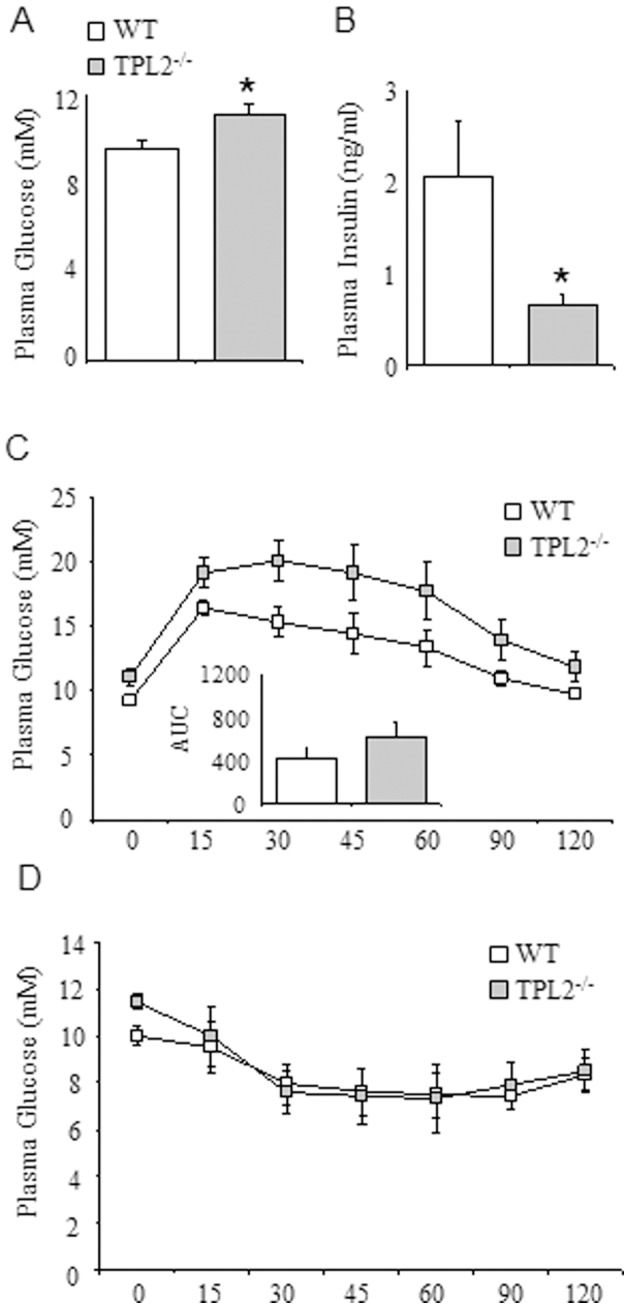
*wt* (n = 8) and *tpl2^−/−^* (n = 8) mice were fed a standard chow diet for 16 weeks. (A) Fasting plasma glucose. (B) Fasting plasma insulin. (C) Glucose tolerance test. (D) Insulin tolerance test. In C, the inset graph is the incremental area under the curve. Data presented are mean ± SEM. * denotes statistically significant difference between *wt* and *tpl2^−/−^*.

Mice fed a HF diet containing 42% calories by energy from fat for 16 weeks were approximately 9 g heavier than standard chow fed animals (Figure 1A), a difference entirely attributable to an increase in fat mass (Figure 1B, C and D). No statistically significant differences in fat mass were observed between the *wt* and *tpl2^−/−^* mice on either the standard chow or HF diet. To examine metabolic control in *wt* and *tpl2^−/−^* mice, we initially determined plasma glucose and insulin levels following a 5 h fast. Fasting plasma glucose levels were significantly higher in *tpl2^−/−^* mice fed a chow diet (Figure 2A). Furthermore, fasting plasma insulin levels were significantly lower in *tpl2^−/−^* mice on a standard chow diet (Figure 2B). We next determined glucose and insulin tolerance in *wt* and *tpl2^−/−^* mice fed a standard chow diet. While we observed the expected increases and decreases in plasma glucose over a 2 hr time course following the IP injection of glucose and insulin respectively, no differences were observed for either glucose or insulin tolerance between the two genotypes (Figures 2C and D). Of note, while plasma glucose levels during the GTT are slightly higher in the *tpl2^−/−^* mice, this effect is entirely attributable to the elevated basal glucose levels observed in these animals. Therefore, when the incremental area under the glucose curves were calculated (Figure 2C; inset), no significant differences were observed in glucose tolerance (p value of 0.38) between the two genotypes.

**Figure 3:**
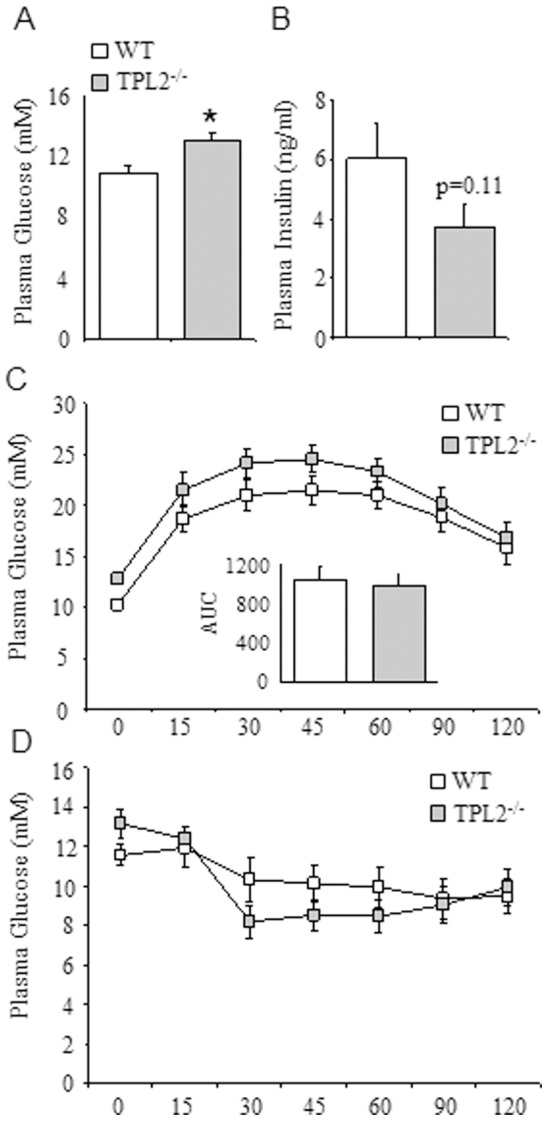
*wt* (n = 7) and *tpl2^−/−^* (n = 8) mice were fed a 42% HF diet for 16 weeks. (A) Fasting plasma glucose. (B) Fasting plasma insulin. (C) Glucose tolerance test. (D) Insulin tolerance test. In C, the inset graph is the incremental area under the curve. Data presented are mean ± SEM. * denotes statistically significant difference between *wt* and *tpl2^−/−^*.

Next, we examined the effects of HF diet (42% calories by energy content derived from fat) on metabolic control in *wt* and *tpl2^−/−^* mice. Consistent with the data observed in the standard chow fed animals, basal plasma glucose levels following a 5 h fast were significantly higher in *tpl2^−/−^* mice compared to *wt* mice (Figure 3A). While fasting plasma insulin levels tended to be lower in the *tpl2^−/−^*mice, this difference did not reach statistical significance (Figure 3B). Importantly, when we determined glucose and insulin tolerance, no significant differences were observed between *wt* and *tpl2^−/−^* mice (Figure 3C and D). Again, similar to our observation in the standard chow fed mice, the plasma glucose levels during the GTT are slightly higher in the *tpl2^−/−^* mice. However, these differences are again attributable to an increase in fasting basal glucose levels and when the incremental area under the glucose curve is calculated (Figure 3C; inset) no differences are observed in glucose tolerance between the genotypes (p value of 0.98).

**Figure 4:**
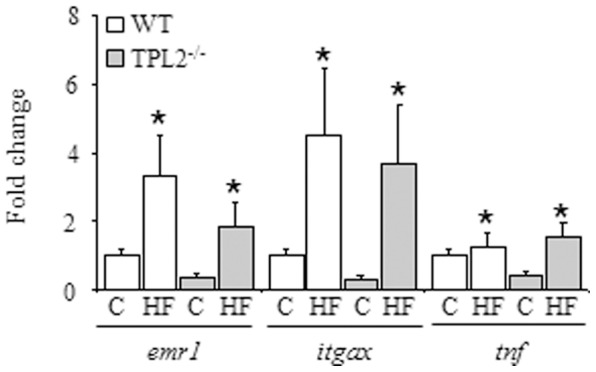
*wt* and *tpl2^−/−^* mice were fed either standard chow or 42% high fat diet for 16 weeks. mRNA expression was determined in epididymal adipose tissue depots. Data are expressed as fold change from *wt*, standard chow fed mice. Data presented are mean ± SEM. (n = 5–6). * denotes statistically significant dietary main effect. C = Standard chow; HF = High Fat.

Our metabolic data strongly argued that Tpl2 plays no role in regulating metabolic responses in either standard chow or HF fed mice. Nonetheless, we performed an analysis of white adipose tissue (epididymal fat depot) to determine the expression of a number of genes that serve as indicators of macrophage recruitment and inflammation, factors that are known to be associated with obesity and implicated in the development of insulin resistance. The expression of *emr1* (also known as F4/80), a general macrophage marker, *itgax* (also known as CD11c), a marker of pro-inflammatory macrophages important in the development of insulin resistance [24], and the expression of the pro-inflammatory cytokine *tnf*, were all significantly increased in HF fed mice, relative to standard chow fed mice (Figure 4). However, no statistically significant differences were observed between *wt* and *tpl2^−/−^* mice on either the standard chow or HF diets.

**Figure 5:**
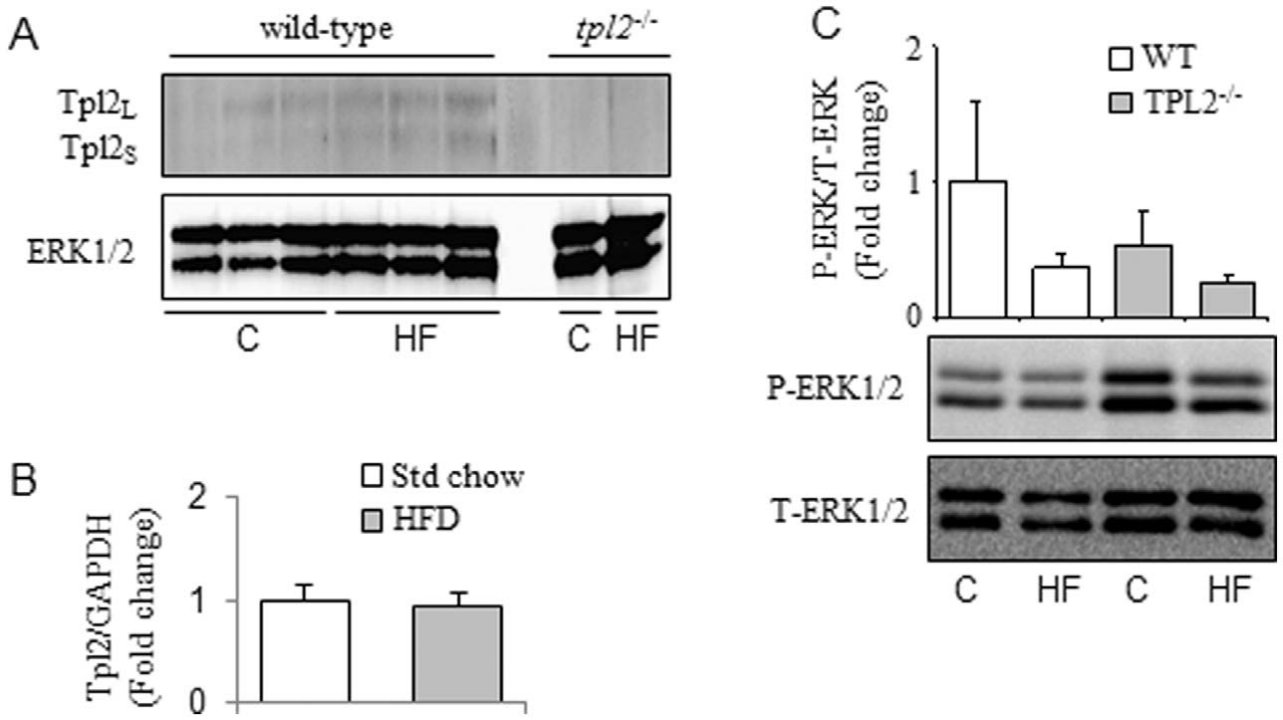
In (A) and (C) *wt* and *tpl2^−/−^* mice were fed either standard chow or 42% high fat diets for 16 weeks. (A) Protein expression was determined by Western blotting in epididymal adipose tissue depots. (B) Tpl2 mRNA expression was determined in *wt* mice that were fed a standard chow (n = 13) or 42% high fat (n = 15) diet for 12 to 16 weeks from 3 independent experiments. (C) Protein expression was determined by Western blotting in epididymal adipose tissue depots. N = 6 in each group. All data presented are mean ± SEM. C = Standard chow; HF = High Fat.

Another published report showed that Tpl2 expression is up-regulated in adipose tissue of mice on a HF diet [18]. Contrary to their findings, we observe similar Tpl2 protein levels and mRNA expression in *wt* mice regardless of the fat content in the diet (Figure 5A and B). As described above, the primary substrates of Tpl2 are MKK1 and MKK2 [17]. Phosphorylation of MKK1/2 by Tpl2 promotes the subsequent phosphorylation and activation of ERK1/2. We, therefore, determined the activation status of ERK1/2, a key target of Tpl2, in the adipose tissue of *wt* and *tpl2* ^−/−^ mice on standard chow and HF diets. We observed no HF diet-induced increases in ERK1/2 phosphorylation (Figure 5C), arguing against a prominent role for Tpl2 in mediating ERK activation in response to diet *in vivo*. This lack of difference between standard chow and HF fed mice demonstrates that the Tpl2 signalling pathway is not activated by HF feeding/obesity and, therefore, argues against a significant role for Tpl2 in obesity-associated metabolic dysfunction.

**Figure 6:**
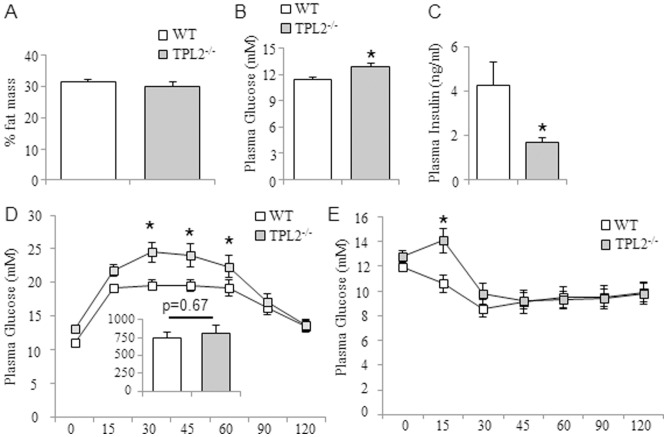
*wt* (n = 10) and *tpl2^−/−^* (n = 9) mice were fed a 59% high fat diet for 16 weeks. (A) Percentage body fat. (B) Fasting blood glucose. (C) Fasting plasma insulin. (D) Glucose tolerance test. (E) Insulin tolerance test. In D, the inset graph is the incremental area under the curve. Data presented are mean ± SEM. In B and C, * denotes statistically significant difference as determined by Student’s T-test. In D and E, * denotes statistically significant difference between *wt* and *tpl2* ^−/−^ at indicated time points as determined by 2-way repeated measures ANOVA. Data presented are mean ± SEM.

In contrast to the results described above, a previous report demonstrated that *tpl2* ^−/−^ mice fed a HF diet containing 60% of calories derived from fat were protected from the development of obesity-associated insulin resistance [19]. A key difference between this previous study and the current work is the fat content of the HF diet, 60% versus 42%. To determine whether differences in the fat content of the HF diet could explain the divergent results between our study and the published report [19], we fed *wt* and *tpl2* ^−/−^ mice a HF diet containing 59% of calories derived from fat. Commencing at approximately 8 weeks age *wt* (n = 10) and *tpl2* ^−/−^ (n = 9) mice were fed a HF diet containing 59% of calories from fat for 16 weeks. This protocol is very similar to that used in the previous study [19] where mice began the HF diet at 6 weeks of age and were fed for 17 weeks. After 16 weeks of HF feeding *wt* and *tpl2* ^−/−^ mice had gained 9.8 g and 8.8 g of fat mass respectively, figures very similar to that achieved with the 42% HF diet (Figure 1C; *wt* 8.6 g, *tpl2* ^−/−^9.0 g). As shown in Figure 6A, the percentage of fat mass at the end of the 16 week HF diet was not different between *wt* and *tpl2* ^−/−^ mice and was very similar to that achieved with the 42% HF diet (Figure 1D). Consistent with the results from the standard chow and 42% HF diet, *tpl2* ^−/−^ mice fed a 59% HF diet displayed fasting hyperglycaemia and hypoinsulinaemia relative to *wt* mice (Figures 6B and C). Similar to the data obtained with the 42% HF diet, *tpl2* ^−/−^ mice fed on the 59% HFD had higher peak blood glucose concentrations at several time points during the GTT compared to *wt* mice (Figure 6D). However, these differences are primarily reflective of the higher basal blood glucose concentration and when the incremental area under the curve is calculated overall glucose tolerance is not different between *wt* and *tpl2* ^−/−^ mice (Figure 6D; inset). Interestingly, when we assessed insulin tolerance, we observed a mild but significant impairment in the ability of insulin to lower blood glucose in the *tpl2* ^−/−^ mice compared to the *wt* mice (Figure 6E).

## Discussion

Given the previously described roles for Tpl2 in regulating responses to various inflammatory stimuli, we hypothesised that Tpl2 deletion may protect against the development of obesity-associated metabolic dysfunction. Using a Tpl-2 deficient mouse strain, our collective findings demonstrate that Tpl2 plays no significant role in the development of the deleterious metabolic consequences associated with a HF diet. Specifically, *tpl2^−/−^* and *wt* mice accrue similar levels of body fat, irrespective of whether they are fed a HF diet containing 42% or 59% of calories from fat, and exhibit comparable glucose and insulin tolerance over the course of a 16 week HF diet. Markers of macrophage recruitment and inflammation following the 42% HF diet, while elevated relative to standard chow fed mice, were comparable between *tpl2^−/−^* and *wt* mice. Finally, similar levels of Tpl2 expression and the lack of ERK1/2 activation in adipose tissue when comparing standard chow and HF fed mice, suggests that the Tpl2 signaling pathway is not activated by high fat feeding-induced obesity. Collectively, our results argue against a non-redundant role for Tpl2 in the development of obesity-associated metabolic dysfunction.

The results of our study are in contrast with a recent report [19] and do not support another study that hypothesised a role for Tpl2 in the adipose tissue dysfunction associated with obesity [18]. One of these previous studies [19] used the same strain of *tpl2^−/−^* mice as that used in the work reported here and, therefore, these phenotypic differences must be attributable to experimental variables that differ between the different studies [19]. In our initial experiments we used a HF diet containing 42% of calories, which contrasted with that used by Perfield and colleagues who used a HF diet containing 60% of calories derived from fat. Comparison of the data obtained in our study using the 42% HF diet and the 60% HF diet used by Perfield and colleagues shows that the effectiveness of these two diets, with respect to the development of obesity, was very comparable. Nonetheless, it is possible that the use of a diet containing significantly more fat may have provided a greater inflammatory stimulus that breached a threshold for Tpl2 activation, thereby revealing a role for Tpl2 in the development of obesity-associated inflammation and insulin resistance. In support of this hypothesis, while we observed no effect of HF feeding on ERK1/2 activation, Perfield et al [19] did report a dietary effect on the status of ERK1/2 phosphorylation. They demonstrated that this HF diet-induced ERK1/2 activation is reduced in the *tpl2^−/−^* mice, suggesting that the increase in ERK1/2 phosphorylation following HF diet is Tpl2 dependent. Therefore, it is possible that in response to a diet containing a very high fat content, Tpl2 signalling may be activated and under such conditions deletion of Tpl2 may protect against the development of obesity-associated dysfunction. To definitively address this issue we performed a second set of experiments using a HF diet containing 59% of calories derived from fat. Consistent with our own data using the 42% HF diet, deletion of Tpl2 did not protect mice from the deleterious metabolic consequences of the HF diet. Instead, there was a trend towards Tpl2 deletion exacerbating the effects of the HF diet, i.e. impaired insulin tolerance, compared to *wt* mice. It is not clear why the results of our study and that of Perfield and colleagues differ. Environmental conditions such as being housed in different animal facilities may potentially explain this discrepancy.

Somewhat unexpectedly, we observed higher fasting plasma glucose levels in both standard chow and 42% and 59% HF fed *tpl2^−/−^* mice when compared with the *wt* controls. Indeed, in the standard chow and 59% HF fed *tpl2^−/−^* mice, we also observed lower fasting plasma insulin levels; a similar trend was also observed on the 42% HF diet. Although these data are consistent with a pancreatic insufficiency, general islet morphology was similar in *wt* and *tpl2^−/−^* mice (Figure S1). More importantly, when challenged with a glucose load, both standard chow and 42% and 59% HF fed *tpl2^−/−^* mice were equally tolerant and cleared the glucose bolus as effectively as *wt* mice arguing against any pancreatic insufficiency in the *tpl2^−/−^* animals. It remains unclear why the *tpl2^−/−^* display basal fasting hyperglycaemia.

In summary, our findings do not support a role for Tpl2 in HF diet-induced metabolic dysfunction. Consistent with this, we do not observe any evidence of activation of the Tpl2 pathway in adipose tissue of HF fed *wt* mice. The conclusions of our study are in contrast to two previous reports that suggest an important role for Tpl2 in mediating obesity-induced inflammation and insulin resistance. Therefore, we believe that a more rigorous analysis on the role Tpl2 in human metabolic syndrome should be carried out before embarking on targeting Tpl2 for improvement of the metabolic state in obesity.

## Materials and Methods

Male *wt* and *tpl2^−/−^* mice were housed in a pathogen-free barrier protected environment on a 12 h:12 h light:dark cycle. All procedures were approved by the Alfred Medical Research and Education Precinct (AMREP) animal ethics committee. This study was specifically approved by the AMREP animal ethics committee (approval no. E/0861/2009/F). *tpl2^−/−^* mice were generated as described previously [12] and were backcrossed more than 12 generations to the C57BL/6 background. C57Bl/6 mice were used as *wt* controls. *wt* and *tpl2^−/−^* mice were fed either standard mouse chow diet containing 8% calories from fat, 21% from protein and 71% from carbohydrate, or a high fat diet containing either 42% calories from fat, 20% from protein and 35% from carbohydrate or a high fat diet containing 59% calories from fat, 15% from protein and 26% from carbohydrate (Speciality Feeds, Glen Forest, Australia). All mice commenced either standard chow or high fat diets at 8 weeks of age.

Body composition was determined using an EchoMRI body composition analyzer (Columbus Instruments, OH, USA). Glucose tolerance tests (GTT) were performed following a 5 h fast. 1 g of glucose per kg of lean body mass, as determined from the EchoMRI analysis, was injected intraperitoneally (IP) and blood glucose concentrations were determined from blood samples obtained from a tail clip. Blood glucose concentrations were determined at baseline and at 15 minute intervals until 1 h and then at 30 minute intervals thereafter. Insulin tolerance tests were performed in an analogous manner to GTT except that 0.75 U (HF diet) or 0.5 U (standard chow diet) insulin per kg of lean body mass was injected. Blood glucose concentrations were determined using an ACCU-CHEK glucose analyzer (Roche). Plasma insulin was determined by enzyme-linked immunosorbant assay (Millipore).

For Western blotting, approximately 50 mg of white adipose tissue from the epididymal fat pad was homogenized in ice cold lysis buffer containing 50 mM TRIS (pH 7.4), 130 mM NaCl, 5 mM EDTA, 1% NP-40, 1 mM PMSF, 1 mM NaF, Phosphatase Inhibitor Cocktail (Containing sodium vanadate, sodium molybdate, sodium tartarate, and imidazole; Sigma-Aldrich) and Protease Inhibitor Cocktail (Containing 4-(2-aminoethyl) benzenesulfonyl fluoride (AEBSF), pepstatinA, E-64, bestatin, leupeptin, and aprotinin; Sigma-Aldrich). Following total protein determination using the BCA assay, lysates were solubilized in Laemelli’s buffer and heated at 95°C for ∼5 min. For Western blotting, 20 to 40 µg of total protein was resolved by SDS-PAGE and transferred to Nitrocellulose membrane (0.2 µm; Bio-Rad Laboratories). Following transfer, membranes were washed (TBS +0.5% Tween; TBST), blocked (TBST containing 5% skim milk) and washed again prior to overnight incubation (4°C) with specific primary antibodies. Following washing, membranes were incubated with HRP-linked secondary antibody (GE Healthcare, UK) for 1 hour at room temperature. After a final wash, membranes were developed using ECL (GE Healthcare, UK). Phospho-ERK antibody was obtained from Cell Signaling Technology (cat#9106). Antibodies against total ERK1/2 (Cat# Sc-94) and Tpl2 (cat# Sc-720) were obtained from Santa Cruz Biotechnology.

For gene expression analysis, total RNA was extracted from epididymal fat depots by TRIZOL extraction performed according to the manufacturer’s instructions. 1.5 µg of RNA was reverse transcribed according to the manufacturer’s instructions (TaqMan Reverse Transcription Reagents; Applied Biosystems). Expression of *emr1*, *itgax*, *tnf* and 18S was determined by real time RT-PCR using specific primer and probe sets (Assays-on-Demand™, Applied Biosystems). Expression of *tpl2* and *gapdh* were determined using specific primers (Tpl2 forward 5′-CTTGCATTTGCAAACCATGC-3′, reverse 5′-GGAACAAGGAGAACATCCGA-3′; GAPDH forward 5′-TTGGCCGTATTGGGCGCCTG-3′, reverse 5′-CACCCTTCAAGTGGGCCCCG-3′) and detected using Fast SYBR Green (Applied Biosystems). All samples were measured in duplicate and data were analysed using the comparative CT method and are expressed as a fold change from *wt* standard chow fed animals.

For histology, pancreata were fixed in 4% paraformaldehyde, cut at a thickness of 5 µm, mounted onto 3-aminopropyltriethoxy-silane (AES)-coated slides and stained with Hematoxylin and Eosin.

All data are expressed as the mean ± SEM. Westerns Blots were quantified using Quantity One software. Data was analyzed by Student’s t-test or ANOVA followed by pairwise comparison testing (Student Newman Keuls Method) where appropriate, using SigmaStat Statistical Software Version 3.5. P-values ≤0.05 were accepted as statistically significant.

## Supporting Information

Figure S1 ***Pancreatic sections from wt*** **and** ***tpl2^−/−^*** **mice fed either a standard chow or high fat diet for 16 weeks were stained with haematoxylin and Eosin.** Data is representative of three mice in each group.
(TIF)
