# Supplementary figures and images for: Tumor Progression Locus 2 (Tpl2) Deficiency Does Not Protect against Obesity-Induced Metabolic Disease
Source: PLoS One. 2012 Jun 11;7(6):e39100. doi: 10.1371/journal.pone.0039100 (PMC3372481; doi:10.1371/journal.pone.0039100)

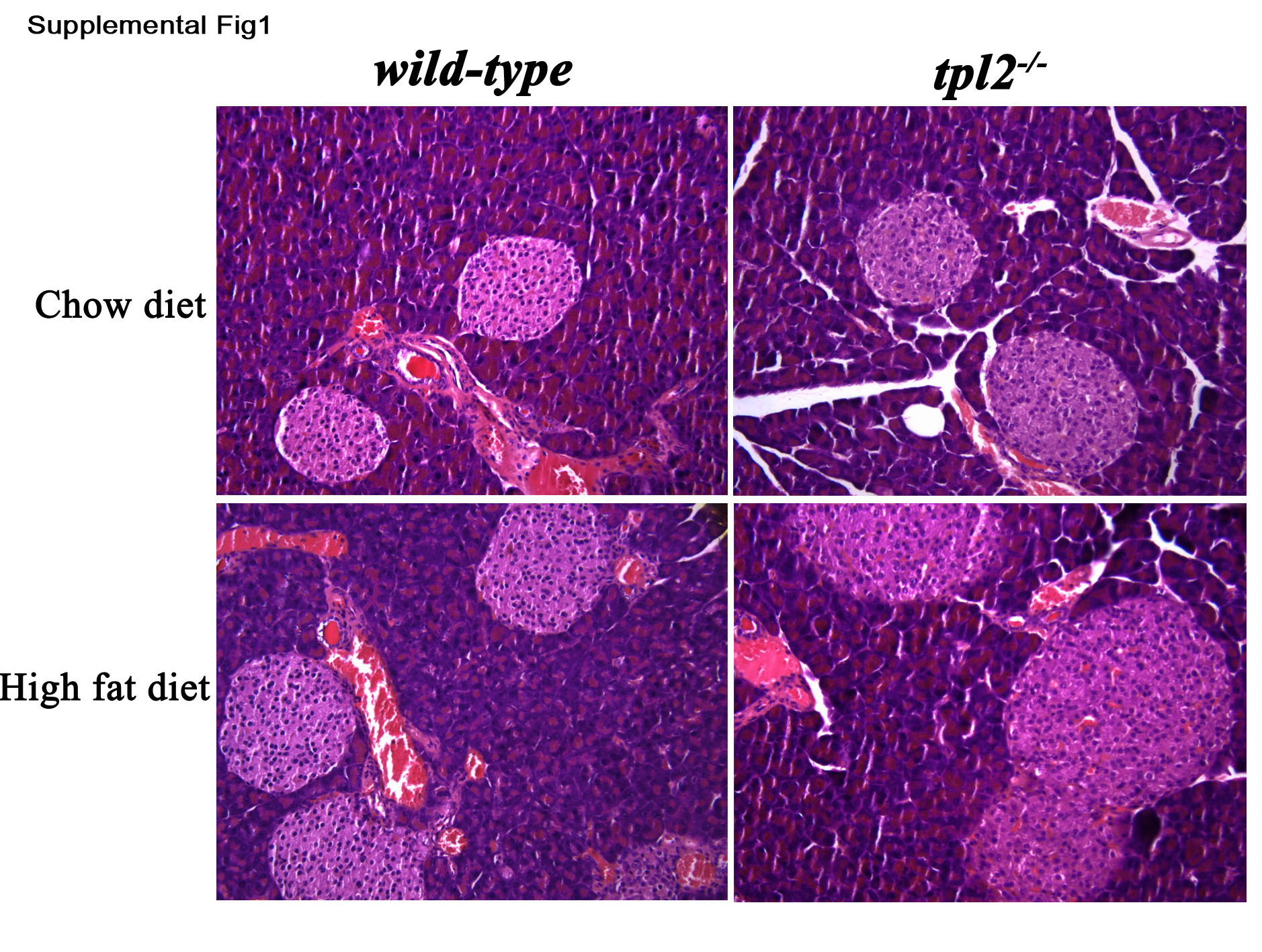

Supplement: Figure S1 — Pancreatic sections from wt and tpl2−/− mice fed either a standard chow or high fat diet for 16 weeks were stained with haematoxylin and Eosin. Data is representative of three mice in each group. (TIF) [file pone.0039100.s001.tif]
